# Urinary Cypermethrin Metabolites among Conventional and Organic Farmers in Thailand

**DOI:** 10.3390/toxics11060507

**Published:** 2023-06-05

**Authors:** Atima Tremongkoltip, Sumate Pengpumkiat, Pornpimol Kongtip, Noppanun Nankongnab, Sukhontha Siri, Susan Woskie

**Affiliations:** 1 Department of Occupational Health and Safety, Faculty of Public Health, Mahidol University, 420/1 Rajvidhi Road, Bangkok 10400, Thailand; atima.tre@gmail.com (A.T.); sumate.pen@mahidol.ac.th (S.P.); noppanun.nan@mahidol.ac.th (N.N.); 2 Department of Epidemiology, Mahidol University, 420/1 Rajvidhi Road, Bangkok 10400, Thailand; sukhontha.sir@mahidol.ac.th; 3 Department of Public Health, University of Massachusetts Lowell, 61 Wilder St., Lowell, MA 01854, USA; susan_woskie@uml.edu

**Keywords:** urinary metabolite, cypermethrin, conventional farmers, organic farmers

## Abstract

Cypermethrin, a pyrethroid insecticide, is frequently spread on agricultural farmlands and is also used in households in Thailand. Conventional pesticide-using farmers (*n* = 209) were recruited from the Phitsanulok and Nakornsawan provinces. Certified organic farmers (*n* = 224) were also recruited in the Yasothorn province. The farmers were interviewed via a questionnaire and the urine from their first morning void was collected. The urine samples were analyzed for 3-phenoxybenzoic acid (3-PBA), cis-3-(2,2-dichlorovinyl)-2,2-dimethylcyclopropane carboxylic acid (cis-DCCA), and trans-3-(2,2-dichlorovinyl)-2,2-dimethylcyclopropane carboxylic acid (trans-DCCA). The results showed no significant difference in the urinary cypermethrin metabolites between the conventional farmers and the organic farmers, for whom the usage of cypermethrin was not accounted for. However, when conventional farmers who used cypermethrin on the farm and in the home were compared with conventional farmers who did not use any cypermethrin or with organic farmers, a significant difference was noted for all metabolites except for trans-DCCA. These findings show that the most significant exposures to cypermethrin are among conventional farmers who use the insecticide on their farms or in their homes. However, measurable levels of all metabolites were found among both conventional and organic farmers who only used cypermethrin in the home or not at all, suggesting that the at-home use of pyrethroids and other possible exposures from pyrethroid residues on market-bought food may contribute to urinary levels of pyrethroids that exceed those of the general population in the US and Canada.

## 1. Introduction

Agriculture is a cornerstone of the economy of Thailand [1]. Many pesticides are currently imported into the country for use in farming [2]. Over 125 pesticides are listed as potential endocrine-disrupting chemicals (EDC) [3,4]. Around the world, chronic exposure to organophosphate and pyrethroid pesticides has been associated with adverse neurological outcomes, including neurodevelopmental delays in children, endocrine-related effects including metabolic disruption, obesity, and diabetes, and reproductive effects such as reduced sperm quality, impacts on reproductive hormones, and pregnancy outcomes such as lower gestational age and birth weight [5,6,7].

Cypermethrin is an insecticide in the pyrethroid group that is often used as an alternative to some of the organophosphate pesticides. It is used in agricultural fields and in households [8,9]. In 2020, 1262 tons of cypermethrin and profenofos were imported into Thailand [2]. Cypermethrin can enter the body mainly through inhalation and skin contact; it is metabolized very quickly by cytochrome P450 into three main metabolites, 3-phenoxybenzoic acid (3-PBA), cis-3-(2,2-dichlorovinyl)-2,2-dimethylcyclopropane carboxylic acid (cis-DCCA), and trans-3-(2,2-dichlorovinyl)-2,2-dimethylcyclopropane carboxylic acid (trans-DCCA), and is then excreted into the urine [10,11]. These metabolites can be used as biomarkers of cypermethrin exposure [12,13]. However, 3-PBA, cis-DCCA, and trans-DCCA are non-specific metabolites of cypermethrin as they are also metabolites of other pyrethroids [12]. For example, 3-PBA was detected in more than 70% of urine samples in the general population of the US and Spain [14,15]. Previously, 3-PBA and cis-trans-DCCA have been detected in the urine samples of conventional pesticide-using farmers in Thailand [16,17]. However, the focus of this study was to evaluate cypermethrin metabolites in the urine of both conventional and organic farmers, while accounting for at-home and occupational usage. 

## 2. Materials and Methods

### 2.1. Study Population and Data Collection

This was a cross-sectional study collecting information from conventional and organic farmers. All subjects gave their informed consent before participating in the study. The study was conducted in accordance with the Declaration of Helsinki, and the protocol was approved by the Ethics Committee of Human Research, Faculty of Public Health, Mahidol University (MUPH 2015-146). All participants signed informed consent forms before participating in the study. We recruited conventional farmers in two provinces in Thailand: rice and vegetable farmers in Phitsanulok province, and sugarcane and rice farmers in Nakornsawan province. One farmer who sprayed his crops with pesticides was selected in each conventional farming household. The organic farmers were certified as using organic farming practices for all their crops. Organic farmers were recruited from rice, vegetable, and fruit farms in the Yasothorn province. One participant who mainly worked on the farm was selected from each organic farming household. Health-promoting hospital staff, community leaders, and hired site officers helped to recruit farmers. The studied farmers could be male or female and 18 years of age or older but they must not have been diagnosed with or be undergoing treatment for diabetes, high blood pressure, heart disease, or thyroid disease. 

A questionnaire was employed to collect data on the demographic characteristics of farmers, risk factors regarding pesticide exposure, home location and home pesticide use, self-reported health problems, agricultural activities, and history of pesticide use. The farmers were interviewed at the health-promoting hospital or in their homes by site officers or trained research team members. The farmers were trained on how to collect a urine sample and were provided with a sterile polyethylene bottle to collect the urine sample. They were asked to collect the first morning void of urine and bring the urine sample to the research team at the health-promoting hospital in their area. The urine samples were kept at −45 °C before analysis.

### 2.2. Urine Sample Analysis

All urine samples were analyzed following the method of Leng and Gries [18] using gas chromatography-mass spectrometry (GC-MS). The calibration curves of cypermethrin metabolites including cis-DCCA, trans-DCCA, and 3-PBA were set up at concentrations ranging from 0 to 100 ng/mL. The accuracy of the 3-PBA analysis ranged from 97.94 to 105.07% at the concentrations of 10 and 60 ng/mL, with a relative standard deviation (RSD) of less than 2. The accuracy of cis- and trans-DCCA was 97.95–106.95% and 95.50–104.11% at the concentrations of 10 and 60 ng/mL, respectively, with an RSD of less than 5. The detection limits of 3-PBA, cis-DCCA, and trans-DCCA were 0.5, 0.5, and 0.5 ng/mL, respectively. For urinary concentrations below the detection limit, we substituted the detection limit by dividing it by the square root of 2 for metabolites where the geometric standard deviation (GSD) was <3, or we substituted the detection limit divided by two for metabolites where the GSD was ≥3 [19]. The creatinine (mg/dL) level in urine was analyzed using an enzymatic method with a linear concentration range of 1–500 mg/dL and a detection limit of 0.16 mg/dL [20]. All urinary metabolites were corrected for creatinine levels by dividing the metabolite concentration by the creatinine concentration, producing units of ng metabolite/g creatinine. This figure was subsequently converted to nmole metabolite/g creatinine for presentation. The results were presented as 3-PBA, cis-DCCA, trans-DCCA, cis,trans-DCCA = cis-DCCA + trans-DCCA, and total cypermethrin = cis,trans-DCCA + 3PBA.

### 2.3. Statistical Analysis

All statistical analyses were performed in SPSS for Windows, version 23 (IBM Thailand Co., Ltd., Bangkok, Thailand). A descriptive analysis of the demographic characteristics was carried out using the chi-square test, Fisher’s exact test, independent *t*-tests, and a one-way ANOVA. Due to the skewed distribution of cypermethrin metabolites, a natural logarithm was used in all analyses.

## 3. Results

The conventional farmers were more commonly male (74.2%) than female (25.8%), while the organic farmers showed a similar percentage of males (51.3%) and females (48.7%) (Table 1). 

The average age and education level of the organic farmers was significantly higher than those of the conventional farmers. The organic farmers were significantly more likely to have a normal BMI and second jobs than the conventional farmers. However, the hours of agricultural work and second job working time were not significantly different. The conventional farmers’ group included significantly more smokers and alcohol drinkers than the organic farmers’ group.

The conventional farmers were significantly more likely than the organic farmers to live near farmland and use insecticides in their homes over the past year (89.5 % vs. 14.8%) (Table 2).

As a result, conventional farmers were also more likely to use the insecticide brands of Ars, Baygon, and Shieldtox, and mosquito coils in their homes over the past year. About 45.9 percent of the conventional farmers used cypermethrin on their farms, while the organic farmers did not use any pesticides on their crops, including cypermethrin. Most of the conventional farmers (57.9%) and organic farmers (66.7%) ate vegetables every day. Sixty-five percent of the conventional farmers did not take a shower after spraying pesticides and 50.2% of conventional farmers kept all their used pesticide containers at home for disposal.

A comparison of the urinary cypermethrin metabolites found that there was no significant difference between the metabolite levels of conventional and organic farmers (Table 3). 

However, among conventional farmers, those who used cypermethrin both on the farm and at home had significantly higher levels of urinary 3PBA, cis-DCCA, cis,trans-DCCA, and total cypermethrin compared to those who only used it in the home (Table 4).

When comparing the samples from conventional farmers who used cypermethrin on their farms and in their homes with the samples from those who did not use cypermethrin in either location, we found that the cis-DCCA level was borderline significantly lower among the non-users. When conventional farmers who used insecticides only at home or not at all were compared with organic farmers who used insecticides only at home or not at all, we found non-significant differences for all cypermethrin metabolites (Table 5).

With regard to organic farmers, the urinary concentrations of cypermethrin metabolites for organic farmers of different ages were not significantly different (Table 6).

Female organic farmers showed significantly higher urinary metabolites of 3-PBA, cis-DCCA, cis,trans-DCCA, and total cypermethrin than male organic farmers. Organic farmers who used insecticides at home in the past year had borderline significantly higher urinary metabolites of cis-DCCA than those who did not use insecticides at home. However, organic farmers who reported the use of Baygon, Shieldtox, or mosquito coils in their homes did not have significantly different urinary metabolite levels compared to those who did not use these home insecticides. Organic farmers who ate vegetables daily had significantly higher urinary 3-PBA levels than those who did not.

For conventional farmers, the urinary concentrations of trans-DCCA, cis,trans-DCCA, and total cypermethrin were significantly higher for those aged 35 to 60 compared to those below the age of 35. The urinary trans-DCCA levels for those over 60 were significantly higher than for those below the age of 35 (Table 7).

Female conventional farmers had significantly higher urinary metabolites of cis-DCCA than male farmers. Farmers who used cypermethrin on their farms had significantly higher 3-PBA, cis-DCCA, trans-DCCA, cis,trans-DCCA, and total cypermethrin levels than those who did not. Farmers who used Baygon in their homes in the past year had significantly higher urinary metabolites of 3-PBA, cis-DCCA, trans-DCCA, cis,trans-DCCA, and total cypermethrin levels than those who did not. Farmers who used ARS in their homes had significantly higher urinary metabolite levels of cis-DCCA than those who did not, while the metabolite levels of those who used Aswin, Shieldtox, and mosquito coils in the home were not significantly different from those who did not. When they sprayed pesticides, those who reported that they did not take a shower or wash immediately after spraying had significantly higher 3-PBA, cis-DCCA, trans-DCCA, cis,trans-DCCA, and total cypermethrin levels than those who did. Conventional farmers who kept all their used pesticide containers at home for disposal had significantly higher 3-PBA, cis-DCCA, cis,trans-DCCA, and total cypermethrin levels than those who did not. Those conventional farmers who ate vegetables daily had significantly higher 3-PBA, cis-DCCA, trans-DCCA, cis,trans-DCCA, and total cypermethrin levels than those who did not report daily vegetable consumption.

## 4. Discussion

Among the conventional farmers, there were more males than females because our inclusion criteria specified that the conventional farmers had to spray pesticides on their own farms [26]. Other studies have reported that most pesticide applicators are male, while female farmers are more likely to choose sowing, hand-picking pests, watering, and harvesting [27]. The organic farmers were older and had a higher level of education than the conventional farmers. This study is similar to that of Chouichom, which showed that longer farm experience (a higher age) and higher education levels were supporting factors in changing over to an organic farming system [28,29]. The organic farmers were more likely to have a normal BMI value than the conventional farmers. This may be because organic farming is more labor-intensive, while conventional farmers use more agricultural machinery in their operations [28,30]. The conventional farmers were significantly more likely to be smokers and alcohol drinkers than the organic farmers. This is probably because there were more male than female conventional farmers and these behaviors are more typical among Thai males [31,32].

Most conventional farmers lived near their farmlands and used insecticides at home; therefore, they may have experienced multiple routes of exposure to pesticides (direct spraying, spray drift, and home use) compared to the organic farmers. The majority of organic farmers (65%) drank bottled water, while the majority (86.6%) of the conventional farmers drank filtered tap water. Pesticide contamination levels in commercial bottled water or in government-provided tap water have not been reported.

There was no significant difference in cypermethrin metabolite levels between the conventional farmers and the organic farmers when cypermethrin usage was not considered. This may be because the conventional farmers generally apply several types of pesticides, including insecticides, herbicides, and fungicides at different times of the year; within these categories, they use a variety of brands [33]. In the current cohort, only 45.9 percent of the conventional farmers reported the use of cypermethrin on their farms. The urinary cypermethrin metabolite levels (3PBA, cis-DCCA, cis,trans-DCCA, and total cypermethrin) from those who used cypermethrin on their farms and in their homes were significantly higher than in samples from those who only used insecticide at home. In most cases, cypermethrin use on both the farm and in the home produced the highest urinary metabolite levels, followed by those who used cypermethrin on the farm only. However, this was generally a non-significant difference, due to the low number of farmers who only used cypermethrin on their crops (*n* = 3). Those who used cypermethrin on their farms could be exposed to cypermethrin during the mixing and spraying of cypermethrin, cleaning and fixing the spraying equipment, and spilling and splashing the cypermethrin solution during spraying [34]. The cypermethrin metabolites found in conventional farmers who used cypermethrin only at home or not at all were not significantly different; this was most likely due to the low number of farmers who did not use cypermethrin at all (*n* = 18) and the high GSD values of the metabolite levels. When we compared the cypermethrin metabolites of conventional farmers and organic farmers who used insecticides only at home or not at all, they were also not significantly different. The similar and non-significant differences between those who only used cypermethrin at home or not at all suggest that the cypermethrin metabolite levels measured may be due to the use of other pyrethroids in the home, from residues on market-purchased foods [17,35,36,37], or from pets treated with insecticides or cleaning products that contain pyrethroids [38,39].

Although we have found no other work that has compared metabolite levels between farmers who used cypermethrin on their farmland and those who used it in their homes, one study looked at pyrethroid exposures among pest-control operators and residents in rural areas. They found that the urinary concentrations of 3-PBA for professional sprayers were significantly higher than for rural residents [40]. In this study, 3-PBA, cis-DCCA, cis,trans-DCCA, and total cypermethrin urinary metabolites among farmers who used cypermethrin on their farms and in their homes were significantly higher than those who only used cypermethrin at home. It is well known that 3-PBA is a non-specific metabolite that is found in many pyrethroids, including cyhalohathrin, cypermethrin, permethrin, deltramethrin, ethonfenprox, esfenvalerate, fenpropathrin, and phenothrin [41]; therefore, if farmers used products containing these agents on their farms or in their homes, the differences that were recorded in 3-PBA levels might be confounded.

Regarding organic farmers, female farmers had significantly higher 3-PBA, cis-DCCA, cis-trans-DCCA, and total cypermethrin levels than male organic farmers. This is probably because female farmers usually take care of the house, cleaning, cooking, and maintaining the home for the whole family, which includes using insecticides at home to get rid of insects, cockroaches, termites, etc. They may, thus, have more contact with the insecticides that are used at home, which mostly contain pyrethroids including cypermethrin, permethrin, cyfluthrin, etc. Organic farmers who reported using insecticides at home in the past year (14.8%), had borderline significantly higher cis-DCCA values than those who were not using them. The organic farmers reported using Aswin (0.6%), ARS (1.7%), Baygon (6.4%), Shieldtox (2.3%), and mosquito coils (10.9%). Most Baygon sprays contain cypermethrin (0.1%) and other pyrethroids, imiprotin (0.03%), and prallethrin (0.03%) [22]. Shieldtox sprays contain other pyrethroids, prallethrin 0.076%, and D-phenothrin 0.046% [23]. Mosquito coils have different brand names with different active ingredients, but the main active ingredients are also pyrethroids, dimefluthrin, allethrin, metofluthrin, etc. [24,25]. When different types of pyrethroids enter the body, they may produce the same or different urinary metabolites. For example, cypermethrin, permethrin, and cyfluthrin have the same urinary metabolites of 3-PBA, cis, and trans-DCCA in humans [41]. Transfluthrin is also metabolized into cis-DCCA, trans-DCCA, and other metabolites, such as 2,3,5,6-tetrafluorobenzyl alcohol and 2,3,5,6-tetrafluorobenzoic acid [42]. The urinary 3-PBA levels of organic farmers who ate vegetables daily were significantly higher than in the samples from those who did not. Vegetables could be sources of exposure for organic farmers if residues persisted after insecticides were used by other farmers who sold their vegetables in the market [43].

With regard to conventional farmers aged 35–60, the concentration levels of trans-DCCA, cis,trans-DCCA, and total cypermethrin were significantly higher than those of farmers below the age of 35. Urinary trans-DCCA levels for those over the age of 60 were significantly higher than for those below the age of 35. Pyrethroids are removed from the body by liver esterase enzymes. As age increases, the liver often loses some functionality, which may be one explanation for the higher metabolite levels recorded among older farmers [44]. It is also possible that older farmers are less careful regarding safe work practices or personal protective equipment when spraying pesticides. This study found that female farmers had higher urinary cis-DCCA levels than male farmers, although none of the other metabolites differed according to gender. A study by Chetna et al. found that female sprayers did not take into account the wind direction while applying pesticides and did not read and understand the pesticide labels, which may explain their higher metabolite levels [45]. Those who used Baygon at home had significantly higher urinary 3-PBA, Cis-DCCA, Trans-DCCA, cis,trans-DCCA, and total cypermethrin levels than those who did not use Baygon at home. This is because Baygon has cypermethrin as its main active ingredient (0.1%) [22]. Those who used ARS had significantly higher urinary Cis-DCCA than those who did not. The main active ingredients of ARS are tetramethrin, permethrin, allethrin, and cypermethrin [21]. Cypermethrin, permethrin, and cyfluthrin produce the same urinary metabolites of 3-PBA, cis-DCCA, and trans-DCCA in humans [41]. We asked the farmers using pesticides whether they took a shower or washed immediately after spraying pesticides; those who said yes demonstrated significantly lower levels of all cypermethrin metabolites than those who did not. When pesticides are mixed, the liquid form is prone to splashing and spillage, resulting in direct or indirect skin contact through clothing contamination [43]. When farmers sprayed the pesticide alachlor, the overspray could be measured on their foreheads, arms, legs, and backs [27]. Showering after spraying can reduce dermal exposure and reduce the pesticide metabolites in the urine. When farmers used up the pesticides, we asked where they kept the empty pesticide bottles. Those who kept the empty bottles at home for disposal had significantly higher 3-PBA, cis-DCCA, cis,trans-DCCA, and total cypermethrin levels than those who did not. The empty pesticide bottles could be a source of pesticide exposure in or around the homes of conventional farmers. The empty pesticide bottles still have residues of pesticides in them. Exposure could occur through the inhalation of residual air concentrations or exposure to the residues found on surfaces, clothing, food, dust, discarded pesticide containers, or application equipment [43]. The cypermethrin metabolites of conventional farmers who ate vegetables daily had significantly higher 3-PBA, cis-DCCA, trans-DCCA, cis,trans-DCCA, and total cypermethrin levels than those who did not. It is important to note that, as described above, the cypermethrin metabolites of organic farmers who ate vegetables daily were significantly higher for only 3-PBA, compared to organic farmers who did not eat vegetables daily. These differences may reflect the finding that the most common source of pesticides is from the vegetables that are being eaten. In the case of conventional farmers, their own or the market vegetables are treated with insecticides. In the case of organic farmers, it is more likely that they mostly ate organic vegetables, with only some supplemental vegetables that could contain pesticide residues from the market. Currently, 3-PBA is a non-specific metabolite of many pyrethroids, including cyhalohathrin, cypermethrin, permethrin, deltramethrin, ethonfenprox, esfenvalerate, fenpropathrin, and phenothrin [41]; finding 3-PBA in the urine of organic farmers suggests that general pyrethroid residues may be found on supplemental market vegetables.

When we compared the concentrations of 3-PBA, cis-DCCA, and trans-DCCA in conventional farmers’ urine to the figures reported in other studies, the metabolite levels measured in this current study were consistent with studies involving farmers, made by Panuwet et al. in Thailand [16] and López-Gálvez et al. in Mexico [44]. These authors studied farmers who applied permethrin and cypermethrin, which can be metabolized into 3-PBA, cis-DCCA, and trans-DCCA. However, the studies by Wang et al. and Kimata et al., regarding pest-control operators in Japan who applied pyrethroid frequently during the summer, reported higher levels of pyrethroid metabolites [40,46]. The pest-control operators in Kimata’s study applied pyrethroid indoors, where there was less ventilation.

For organic farmers, the concentration levels of 3-PBA and trans-DCCA were comparable to those reported in the study conducted by Qi et al. in China. Our study reported that organic farmers used insecticides at home, which was similar to the findings in Qi’s study of pregnant women, where, although they were not exposed occupationally, about half of them reported applying indoor insecticides [47]. However, the levels in our study were higher compared to the general adult populations of the US [48] and Canada [49], where insecticides are used less frequently in the home. These results and those from the conventional farmers who did not use cypermethrin in the home or on their farms suggest other sources of exposure, such as food purchased at the market.

The limitations of this study include the fact that there was only one spot-checked urine sample for each subject. We did not collect data regarding the last time that they applied any specific insecticides on their crops or in the home. The use of cypermethrin on the farm depends on the season and the types of crops, while the use of insecticides in the home also varies with the season. The cypermethrin metabolites have very short half-lives in human urine (3BPA (5.7 h) and DCCA (4.5–5.4 h)) [50]; thus, reports of usage may not have been linked closely enough in time regarding the collection of urine samples to show strong associations. We did not collect information on whether the farmers had pets or animals at home, or whether the farmers used showering or cleaning products such as Chaingard, which contains permethrin (0.5–1%) or cypermethrin (0.1–0.15%), on their pets or animals [51]. Nor did we ask about how much of their food was organic versus how much was not organic. All of these activities could be additional sources of exposure.

## 5. Conclusions

Conventional farmers who use cypermethrin on their farms showed the highest levels of associated metabolites, while both organic and conventional farmers who only used cypermethrin at home or who did not use any cypermethrin demonstrated the lowest urinary levels. Nevertheless, the finding that the levels of these metabolites among organic farmers and conventional farmers who do not use cypermethrin exceeded those in the general population of Western countries suggests that other sources, such as market produce, pets, cleaning products, or the use of other pyrethroids may be contributing to this higher background level.

## Figures and Tables

**Table 1:** Demographic characteristics of conventional (*n* = 209) and organic farmers (*n* = 224).

Variables		Conventional Farmers *n* (%)	Organic Farmers *n* (%)	*p*-Value
Sex ^1^				
	Male	155 (74.2)	115 (51.3)	<0.001 *
	Female	54 (25.8)	109 (48.7)	
Age ^3^				
	Mean (SD)	50.10 (11.1)	53.22 (10.3)	0.003 *
	Min–max	18–69	28–79	
Education level ^1^				
	Below elementary	14 (6.7)	4 (1.8)	0.036 *
	Elementary	119 (56.9)	125 (55.8)	
	High school	71 (34.0)	84 (37.5)	
	Bachelor’s degree or higher	5 (2.4)	11 (4.9)	
Marital status ^2^				
	Single	21 (10.0)	13 (5.8)	0.027 *
	Married	180 (86.1)	188 (83.9)	
	Widowed/divorced	8 (3.8)	23 (10.3)	
Body mass index (BMI) (kg/m^2^) ^1^				
	Normal (<18.49–24.99)	118 (56.7)	167 (74.6)	<0.001 *
	Abnormal (>25.00)	90 (43.3)	57 (25.4)	
	Missing	1 (0.2)		
Expense adequacy ^1^				
	Not in debt	135 (64.6)	143 (63.08)	0.870
	In debt	74 (35.4)	81 (36.2)	
Agricultural work time (h/week) ^3^				
	Mean (SD)	26.5 (3.4)	28.8 (17.2)	0.149
Has a second job ^1^				
	Yes	160 (76.6)	96 (42.9)	<0.001 *
	No	49 (23.4)	128 (57.1)	
Second job work time (h/week) ^3^				
	Mean (SD)	24.9 (13.6)	26.6 (17.8)	0.537
Main types of crop plant				
	Rice ^1^	162 (77.5)	213 (96.8)	<0.001 *
	Sugarcane ^1^	80 (38.3)	14 (6.6)	<0.001 *
	Banana ^1^	14 (6.7)	118 (55.9)	<0.001 *
	Vegetables ^1^	78 (37.3)	180 (84.8)	<0.001 *
	Fruits ^1^	10 (4.8)	102 (48.6)	<0.001 *
Smoking ^1^				
	Current smoker	57 (27.3)	36 (16.1)	0.001 *
	Former smoker	9 (4.3)	25 (11.2)	
	Non-smoker	143 (68.4)	163 (72.8)	
Alcohol intake ^1^				
	Current drinker	134 (64.1)	92 (41.1)	<0.001 *
	Former drinker	7 (3.3)	45 (20.1)	
	Non-drinker	68 (32.5)	87 (38.8)	

^1^ chi-square test, ^2^ Fisher’s exact test, ^3^ independent *t*-test, * significant at a *p*-value < 0.05.

**Table 2:** Risk factors of conventional (*n* = 209) and organic farmers (*n* = 224).

Risk Factors		Conventional Farmers *n* (%)	Organic Farmers *n* (%)	*p*-Value
Living near farm ^1^				
	Yes	177 (84.7)	103 (46.4)	<0.001 *
	No	32 (15.3)	117 (52.7)	
Insecticide use at home in the past 1 year ^1^				
	Yes	187 (89.5)	33 (14.8)	<0.001 *
	No	22 (10.5)	190 (85.2)	
Type of insecticide used at home				
Aswin or Atsawin ^1,2^				
	Yes	2 (1.1)	1 (0.6)	1.000
	No	187 (98.9)	170 (99.4)	
Ars ^1,2^				
	Yes	25 (13.2)	3 (1.7)	<0.001 *
	No	164 (86.8)	169 (98.3)	
Baygon ^1,2^				
	Yes	107 (56.6)	11 (6.4)	<0.001 *
	No	82 (43.4)	161 (93.6)	
Shieldtox ^1,2^				
	Yes	25 (13.2)	4 (2.3)	<0.001 *
	No	164 (86.8)	169 (97.7)	
Mosquito coil ^1,2^				
	Yes	148 (78.3)	19 (10.9)	<0.001 *
	No	41 (21.7)	156 (89.1)	
Cypermethrin used in farm ^1^				
	Yes	95 (45.9)	0 (0)	<0.001 *
	No	113 (54.1)	224 (100)	
Water consumption source ^1^				
	House and community well	9 (4.3)	6 (2.7)	<0.001 *
	Bottled water	14 (6.7)	142 (64.5)	
	Rainwater	5 (2.4)	64 (29.1)	
	Filtered tap water	181 (86.6)	8 (3.6)	
Daily vegetable intake ^1^				
	Yes	121 (57.9)	148 (66.7)	0.060
	No	88 (42.1)	74 (33.3)	
Did not take a shower after spraying ^1^				
	Yes	137 (65.5)	0 (0)	<0.001 *
	No	72 (34.4)	224 (100)	
Keeping all used containers at home for disposal ^1^				
	Yes	105 (50.2)	0 (0)	<0.001 *
	No	104 (49.8)	224 (100)	

^1^ chi-square test, * significant at a *p*-value < 0.05. ^2^ Main ingredients of insecticide used at home. Aswin sprays contain tetramethrin 0.2%, permethrin 0.1% [21]. ARS sprays contain imiprothrin 0.2%, cypermethrin 0.1% [21]. Baygon sprays contain cypermethrin (0.1%) and other pyrethroids, imiprotin (0.03%), and prallethrin (0.03%) [22]. Shieldtox sprays contain other pyrethroids, prallethrin 0.076%, and D-phenothrin 0.046% [23]. Mosquito coils have different brands with different active ingredients, but the main active ingredients are also pyrethroids, dimefluthrin, allethrin, metofluthrin, etc. [24,25].

**Table 3:** Urinary cypermethrin metabolites (geometric mean (GM), geometric standard deviation (GSD), median, nmole/g creatinine), compared between conventional (*n* = 209) and organic farmers (*n* = 224).

Cypermethrin Metabolites	Conventional Farmers (nmole/g Creatinine)		Organic Farmers (nmole/g Creatinine)		*p*-Value ^1^
	GM (GSD)	Median	GM (GSD)	Median	
3-PBA	6.01 (3.31)	6.42	5.12 (3.13)	4.74	0.159
Cis-DCCA	17.78 (2.46)	16.08	15.45 (2.54)	12.48	0.112
Trans-DCCA	11.15 (2.40)	10.7	10.03 (2.48)	8.14	0.219
Cis,Trans-DCCA	30.03 (2.32)	26.65	26.33 (2.45)	21.49	0.113
Total Cypermethrin	37.30 (2.36)	33.78	32.30 (2.46)	26.72	0.089

^1^ *t*-test on the natural logs of metabolite concentration.

**Table 4:** Urinary cypermethrin metabolites (GM (GSD), nmole/g creatinine) of conventional farmers, classified by insecticide use.

	1. Farm and Home Use (*n* = 93)	2. Farm Use Only (*n* = 3)	3. Home Use Only (*n* = 95)	4. No Farm or Home Use (*n* = 18)	*p*-Value of Overall ANOVA	*p*-Value for Multiple Comparisons ^1^
3PBA	9.58 (2.62)	17.93 (4.38)	3.69 (3.41)	5.88 (3.06)	<0.001	(1–3) * *p* < 0.001
Cis-DCCA	24.63 (2.27)	17.58 (2.97)	13.51 (2.33)	14.10 (2.92)	<0.001	(1–3) * *p* < 0.001, (1–4) *p* = 0.059
Trans-DCCA	12.86 (2.36)	9.97 (4.21)	9.86 (2.34)	10.34 (2.68)	0.212	
Cis,Trans-DCCA	38.19 (2.23)	27.85 (3.38)	24.54 (2.21)	25.85 (2.32)	0.003	(1–3) * *p* = 0.002
Total Cypermethrin	48.60 (2.23)	46.06 (3.76)	29.24 (2.26)	33.13 (2.59)	0.001	(1–3) * *p* < 0.001

^1^ Multiple comparisons with the Tukey on natural logs of metabolite concentration, * significant at a *p*-value < 0.05.

**Table 5:** Urinary cypermethrin metabolites (GM (GSD), nmole/g creatinine) of conventional and organic farmers, classified by insecticide use.

	Conventional Farmers		Organic Farmers		*p*-Value of Overall ANOVA *
	Home Use Only (*n* = 95)	No Farm or Home Use (*n* = 18)	Home Use Only (*n* = 33)	No Farm or Home Use (*n* = 191)	
3PBA	3.69 (3.41)	5.88 (3.06)	6.72 (1.25)	4.89 (1.08)	0.045
Cis-DCCA	13.51 (2.33)	14.10 (2.92)	20.66 (1.21)	14.69 (1.07)	0.146
Trans-DCCA	9.86 (2.34)	10.34 (2.68)	10.89 (1.20)	9.89 (1.07)	0.945
Cis,Trans-DCCA	24.54 (2.21)	25.85 (2.32)	32.69 (1.20)	25.37 (1.06)	0.420
Total Cypermethrin	29.24 (2.26)	33.13 (2.59)	40.29 (1.20)	31.09 (1.06)	0.337

* No subgroup multiple comparisons were significant.

**Table 6:** Urinary cypermethrin metabolites (GM (GSD), nmole/g creatinine) of organic farmers, classified by age, sex, insecticide use at home in the past 1 year, home use of insecticides (Aswin, Ars, Baygon, Shieldtox, and mosquito coils), and daily vegetable intake.

	3-PBA		Cis-DCCA		Trans-DCCA		Cis,trans-DCCA		Total Cypermethrin	
	GM (GSD)	*p*-Value	GM (GSD)	*p*-Value	GM (GSD)	*p*-Value	GM (GSD)	*p*-Value	GM (GSD)	*p*-Value
Age ^1^										
1. <35	4.52 (1.45)	0.129	9.42 (1.58)	0.073	7.94 (1.66)	0.311	18.54 (1.33)	0.185	25.15 (1.37)	0.170
2. 35–60	5.57 (3.28)		16.63 (2.68)		10.54 (2.60)		27.89 (2.58)		35.44 (2.56)	
3. >60	3.85 (2.75)		12.53 (2.09)		8.59 (2.15)		22.25 (2.04)		27.94 (2.07)	
Sex ^2^										
Male	3.91 (2.97)	<0.001 *	13.13 (2.38)	0.007 *	8.99 (2.41)	0.064	23.02 (2.30)	0.021 *	27.66 (2.29)	0.008 *
Female	6.81 (3.11)		18.35 (2.65)		11.26 (2.54)		30.34 (2.56)		38.04 (2.56)	
Insecticide use at home in the past 1 year ^2^										
Yes	6.72 (3.59)	0.145	20.66 (3.00)	0.055	10.89 (2.79)	0.584	32.69 (2.84)	0.138	40.29 (2.84)	0.131
No	4.91 (3.05)		14.72 (2.45)		9.91 (2.44)		25.43 (2.38)		31.18 (2.38)	
Type of insecticide used at home										
Baygon ^2,3^										
Yes	6.67 (3.58)	0.607	18.83 (2.86)	0.544	10.45 (2.92)	0.924	29.69 (2.82)	0.723	36.93 (2.87)	0.716
No	5.56 (3.07)		15.68 (2.61)		0.16 (2.53)		26.79 (2.51)		33.25 (2.50)	
Shieldtox ^2,3^										
Yes	6.27 (2.28)	0.839	18.04 (1.54)	0.785	10.78 (2.25)	0.899	29.52 (1.70)	0.841	36.32 (1.73)	0.854
No	5.58 (3.11)		15.79 (2.64)		10.15 (2.55)		26.86 (2.54)		33.32 (2.53)	
Mosquito coil ^2,3^										
Yes	7.12 (3.75)	0.320	21.59 (3.30)	0.137	11.72 (2.85)	0.503	34.72 (3.07)	0.208	43.06 (3.05)	0.205
No	5.43 (2.99)		15.27 (2.51)		10.07 (2.50)		26.20 (2.44)		32.47 (2.43)	
Daily vegetable intake ^2^										
Yes	9.18 (2.30)	0.011 *	15.56 (2.68)	0.196	9.19 (2.95)	0.316	25.47 (2.69)	0.226	35.37 (2.54)	0.114
No	6.66 (2.03)		13.14 (2.31)		8.31 (2.50)		22.24 (2.27)		29.34 (2.17)	

^1^ Multiple comparisons with the Tukey on the natural logs of metabolite concentration, ^2^ *t*-test on the natural logs of metabolite concentration. ^3^ Main ingredients of insecticide used at home. Baygon sprays contain cypermethrin (0.1%) and other pyrethroids, imiprotin (0.03%), and prallethrin (0.03%) [22]. Shieldtox sprays contain other pyrethroids, prallethrin 0.076%, and D-phenothrin 0.046% [23]. Mosquito coils are of different brands with different active ingredients, but the main active ingredients are also pyrethroids, dimefluthrin, allethrin, metofluthrin, etc. [24,25]. * Significant at a *p*-value < 0.05.

**Table 7:** Urinary cypermethrin metabolites (GM(GSD), nmole/g creatinine) of conventional farmers, classified by age, sex, insecticide use at home in the past 1 year, cypermethrin use on their farm, and the use of insecticides at home (Aswin, Ars, Baygon, Shieldtox, and the mosquito coil) did not report taking a bath immediately after spraying, kept all used chemical containers at home for disposal, and had a daily intake of vegetables.

	3-PBA		Cis-DCCA		Trans-DCCA		Cis,trans-DCCA		Total Cypermethrin	
	GM (GSD)	*p*-Value	GM (GSD)	*p*-Value	GM (GSD)	*p*-Value	GM (GSD)	*p*-Value	GM (GSD)	*p*-Value
Age ^1^										
1. <35	4.14 (3.30)	0.287	12.07 (2.50)	0.090	6.82 (2.52)	0.016, (1–2) *p* = 0.013, (1–3) *p* = 0.046	20.16 (2.26)	0.053, (1–2) *p* = 0.042	24.94 (2.33)	0.056,
2. 35–60	6.33 (3.25)		18.76 (2.41)		11.84 (2.41)		31.74 (2.31)		39.45 (2.33)	(1–2) *p* = 0.044
3. >60	6.09 (3.58)		18.22 (2.57)		11.88 (2.13)		30.96 (2.29)		38.15 (2.38)	
Sex ^2^										
Male	5.67 (3.41)	0.241	16.35 (2.50)	0.022 *	10.87 (2.47)	0.477	28.46 (2.36)	0.112	35.32 (2.40)	0.119
Female	7.08 (3.02)		22.61 (2.25)		12.00 (2.22)		35.18 (2.19)		43.63 (2.20)	
Insecticide use at home in the past 1 year ^2^										
Yes	5.89 (3.33)	0.480	18.11 (2.26)	0.389	11.21 (2.37)	0.791	30.43 (2.29)	0.544	37.46 (2.33)	0.831
No	7.13 (3.26)		15.21 (2.84)		10.63 (2.73)		27.11 (2.66)		35.94 (2.64)	
Cypermethrin use on the farm ^2^										
Yes	9.77 (2.66)	<0.001 *	24.37 (2.28)	<0.001 *	12.76 (2.39)	0.040 *	37.82 (2.25)	<0.001 *	48.52 (2.33)	<0.001 *
No	3.97 (3.38)		13.60 (2.41)		9.94 (2.38)		24.74 (2.27)		29.83 (2.30)	
Type of insecticide used at home										
ARS ^2,3^										
Yes	9.24 (2.54)	0.052	25.31 (2.18)	0.043 *	12.46 (2.27)	0.515	38.02 (2.18)	0.151	48.04 (2.17)	0.123
No	5.58 (3.45)		17.22 (2.44)		11.02 (2.41)		29.41 (2.31)		36.20 (2.36)	
Baygon ^2,3^										
Yes	8.03 (2.93)	<0.001 *	21.02 (2.46)	0.008 *	12.54 (2.36)	0.043 *	34.74 (2.29)	0.012 *	43.83 (2.31)	0.004 *
No	4.05 (3.57)		14.92 (2.30)		9.67 (2.40)		25.59 (2.25)		30.75 (2.30)	
Shieldtox ^2,3^										
Yes	5.89 (3.61)	0.958	17.12 (2.46)	0.734	11.63 (2.49)	0.816	29.98 (2.34)	0.924	36.94 (2.42)	0.914
No	5.98 (3.33)		18.27 (2.43)		11.14 (2.38)		30.49 (2.30)		37.68 (2.34)	
Mosquito coil ^2,3^										
Yes	6.09 (3.29)	0.662	18.69 (2.43)	0.358	11.61 (2.37)	0.289	31.57 (2.29)	0.245	38.86 (2.32)	0.307
No	5.54 (3.64)		16.18 (2.41)		9.89 (2.46)		26.61 (2.33)		33.31 (2.43)	
Did not take a bath immediately after spraying ^2^										
Yes	11.12 (2.20)	<0.001 *	25.32 (2.25)	<0.001 *	13.69 (2.32)	0.014 *	40.07 (2.18)	<0.001 *	52.01 (2.15)	<0.001 *
No	4.34 (3.49)		14.77 (2.44)		10.01 (2.41)		25.85 (2.31)		31.32 (2.35)	
Kept all used chemical containers at home for disposal ^2^										
Yes	9.25 (2.48)	<0.001 *	22.44 (2.27)	<0.001 *	12.34 (2.30)	0.092	35.41 (2.22)	0.005 *	45.42 (2.22)	<0.001 *
No	3.88 (3.66)		14.06 (2.52)		10.06 (2.49)		25.49 (2.36)		30.57 (2.40)	
Daily vegetable intake ^2^										
Yes	7.25 (3.04)	0.007 *	20.28 (2.47)	0.013 *	12.40 (2.39)	0.039 *	34.16 (2.30)	0.010 *	42.65 (2.32)	0.008 *
No	4.63 (3.56)		14.84 (2.39)		9.63 (2.38)		25.22 (2.28)		31.02 (2.33)	

^1^ Multiple comparisons with the Tukey scores on the natural logs of metabolite concentration; *p*-value refers to the overall ANOVA and *p*-value of each subgroup; ^2^ *t*-test on the natural logs of metabolite concentration. ^3^ Main ingredients of insecticide used at home. ARS sprays contain imiprothrin 0.2%, cypermethrin 0.1% [21]. Baygon sprays contain cypermethrin (0.1%) and other pyrethroids, imiprotin (0.03%), and prallethrin (0.03%) [22]. Shieldtox sprays contain other pyrethroids, prallethrin 0.076%, and D-phenothrin 0.046% [23]. Mosquito coils have different brands with different active ingredients, but the main active ingredients are also pyrethroids, dimefluthrin, allethrin, metofluthrin, etc. [24,25]. * Significant at a *p*-value < 0.05.

## Data Availability

The data presented in this study are available on request from the corresponding author.
